# Generation of 3D human gastrointestinal organoids: principle and applications

**DOI:** 10.1186/s13619-020-00040-w

**Published:** 2020-06-10

**Authors:** Mengxian Zhang, Yuan Liu, Ye-Guang Chen

**Affiliations:** The State Key Laboratory of Membrane Biology, Tsinghua-Peking Center for Life Sciences, School of Life Sciences, Tsinghua University, Beijing, 100084 China

**Keywords:** Organoids, Gastrointestinal tract, Stomach, Intestine, 3D culture

## Abstract

The stomach and intestine are important organs for food digestion, nutrient absorption, immune protection and hormone production. Gastrointestinal diseases such as cancer and ulcer are big threats to human health. Appropriate disease models are in sore need for mechanistic understanding and drug discovery. Organoids are three-dimensional in vitro cultured structures derived from tissues and pluripotent stem cells with multiple types of cells and mimicking in vivo tissues in major aspects. They have a great potential in regenerative medicine and personalized medicine. Here, we review the major signaling pathways regulating gastrointestinal epithelial homeostasis, summarize different methods to generate human gastrointestinal organoids and highlight their applications in biological research and medical practice.

## Background

The human digestive system is comprised of the mouth, esophagus and gastrointestinal (GI) tract, plus the accessory organs that aid in digestion (Johansson et al., 2013). The GI tract responsible for the food digestion and nutrient absorption includes the stomach, the small intestine and the large intestine. Disorders in the stomach and intestine can lead to various GI diseases such as gastric and colorectal cancers (Bijlsma et al., 2017), gastric ulcer (Graham, 2014) and inflammatory bowel disease (Danese and Fiocchi, 2011; Maloy and Powrie, 2011). These diseases can bring heavy economic burden, lead to poor quality of life and even death. Deep understanding of the disease-causing factors would effectively treat the diseases. During the last few decades, a great effort has been made to understand morphogenesis and homeostasis of the digestive system with various animal models (Eichele and Kharbanda, 2017; Gonzalez et al., 2015; Yin et al., 2017; Zhao and Pack, 2017). However, under some circumstance, species differences in embryonic development and architecture of the adult digestive organs are nonnegligible, which make animal models suboptimal for studying digestive diseases. Till now, the underlying pathogenetic mechanisms of many GI diseases remain ill-defined, and the efficacious therapeutic treatments are unavailable. For decades, however, developing in vitro human disease models had been a great challenge.

In the last decade, human tissue/cancer-derived organoids have attracted a great attention as disease models (Clevers, 2016; Date and Sato, 2015; Lancaster and Knoblich, 2014; Rossi et al., 2018; Tuveson and Clevers, 2019). Organoids are in vitro cultured three-dimensional (3D) cell aggregates derived from stem cells that are capable of self-renewal and self-organization and exhibit certain tissue structures and functionality. Back to 1975, Rheinwatd and Green found that human skin cells could grow from single cells into colonies with a multilayered structure (Rheinwald and Green, 1975). However, a poor understanding of extracellular matrix biology obstructed further research of the 3D culture. It was until 2009 that the organoid research came into a new era. Sato and his colleagues established a brand new Matrigel-based 3D culture system (Sato et al., 2009). Single crypt isolated from mouse intestine can generate a 3D epithelial mini-gut with the addition of various niche factors. The cultured mini-guts share many characteristics with in vivo gut and can generate crypt-like bud structure.

Since the 3D culture system for murine intestinal organoids was set up, different types of organoids originating from various tissues have been generated from adult stem cells or pluripotent stem cells (Dedhia et al., 2016; Fordham et al., 2013; Lancaster et al., 2013; Sato et al., 2011; Takasato et al., 2014). Generous donation of surgically resected tissues or endoscopic biopsy samples contributes a lot to human GI organoid research. In vitro culture of the organoids derived from the human GI tract provides the possibility to study the morphogenesis of the stomach and intestine. More importantly, GI organoids can model diverse digestive diseases and can be used for drug screen and regenerative medicine. This review summarizes the basic knowledge of epithelial homeostasis in GI tract, different methods to generate human GI organoids and their applications and limitations.

## Homeostatic regulation of the gastric and intestinal epithelia

Stomach and intestine both contain a simple epithelium and epithelial homeostasis is vital for their normal function. The successful culture of organoids requires incipient stem cells with an appropriate culture environment. Understanding of the mechanisms underlying epithelial homeostasis in the stomach and intestine is conducive to generate an appropriate organoid culture system.

### Gastric epithelium

**Fig. 1:**
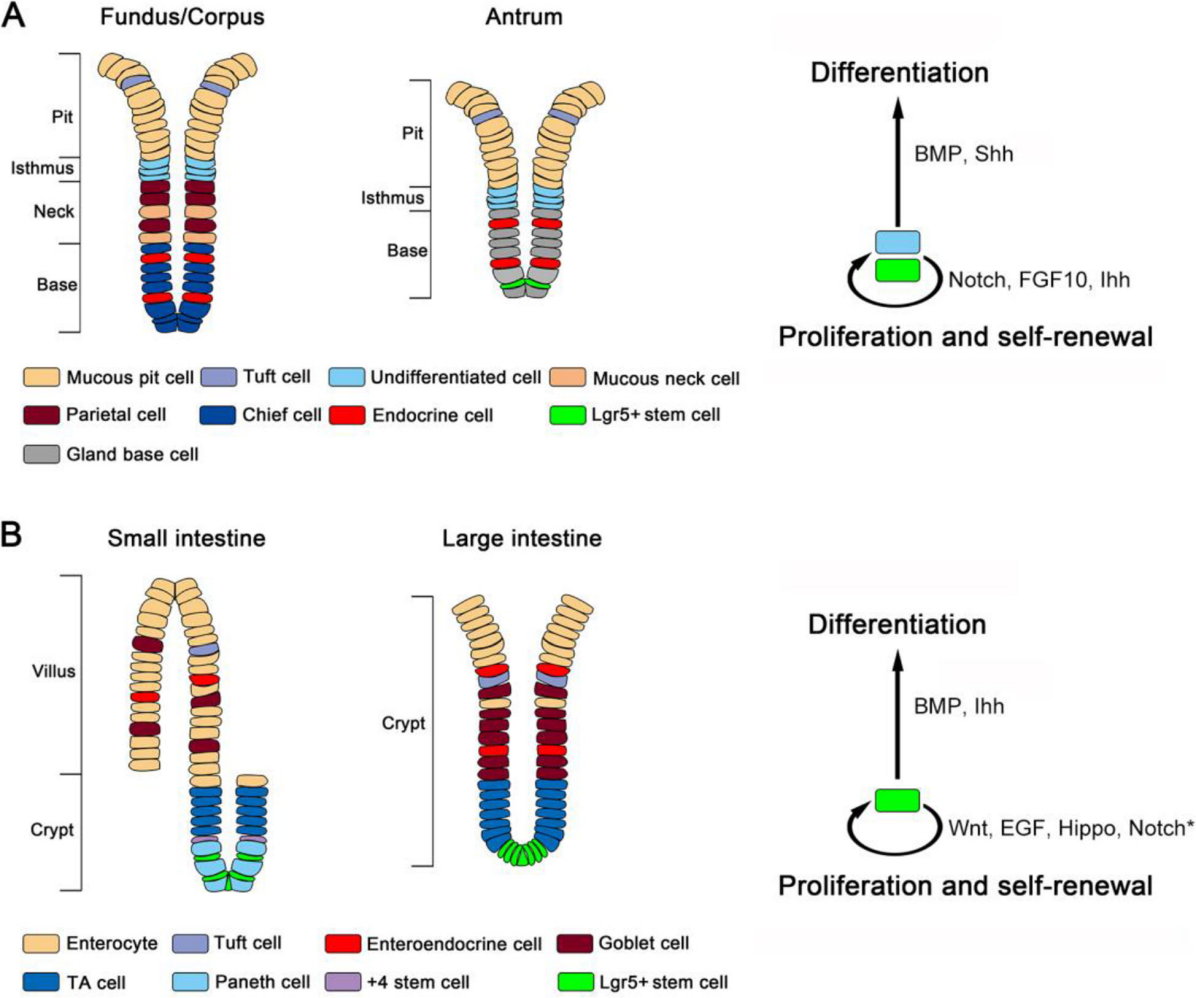
Epithelial homeostasis in stomach and intestine. **a** Left: Schematic diagram of glands in gastric corpus and gastric antrum. Right: Major signaling pathways controlling stem cell homeostasis in stomach. **b** Left: Schematic diagram of small intestinal crypt-villus subunit and large intestinal crypt. Right: Major signaling pathways controlling stem cell homeostasis in intestine. *Notch signaling is also crucial for the secretory lineage differentiation

The main function of stomach is food digestion. The human stomach is anatomically composed of the cardia fundus, corpus and antrum, all of which contains an invaginated single-layer cell glandular epithelium that secretes water, electrolytes, pepsinogen, hydrochloric acid and hormones such as gastrin (Kim and Shivdasani, 2016). The epithelium of the fundus and corpus is comprised of the pit, isthmus, neck and base regions while the antrum epithelium contains the pit, isthmus and base regions (Fig. 1a). Till now the identity of stem cells in the gastric epithelium is still unclear. The isthmus region contains several undifferentiated cells, which have been regarded as gastric stem cells. Indeed, in the corpus, the undifferentiated isthmus cells can give rise to six functional cell types: mucus-producing pit cells, mucus-producing neck cells, acid-secreting parietal cells, endocrine cells, pepsinogen-secreting chief cells, and rare tuft cells. In the antrum, the undifferentiated isthmus cells can differentiate to mucous pit cells, gland base cells, endocrine, cells and tuft cells (Willet and Mills, 2016). However, lineage tracing experiments revealed that Lgr5^+^ cells in the base region of the antrum are self-renewing, multipotent stem cells, responsible for the renewal of the gastric epithelium (Barker et al., 2010). In the corpus, Lgr5^+^ chief cells and Troy^+^ chief cells can also play a role as stem cells after mucosal injury (Leushacke et al., 2017; Stange et al., 2013). Therefore, it is possible that several types of cells contribute to the self-renewal of the gastric epithelium or different types of stem cells function in different parts of the gastric epithelium.

Understanding of homeostatic regulation of the human gastric epithelium is limited, but investigations with the mouse models provide some insights. The homeostasis of the gastric epithelium is regulated by multiple signaling pathways. Notch signaling is important for isthmus cell proliferation. Ectopic Notch expression represses differentiation and induces parietal cells to undifferentiated cells (Kim and Shivdasani, 2011). Fibroblast growth factor 10 (FGF10) promotes epithelial proliferation and inhibits differentiation of parietal cells and chief cells although these effects may not be caused through FGFR2b (Speer et al., 2012). Disruption of bone morphogenetic protein (BMP) signaling in noggin transgenic mice increases cell proliferation and reduces parietal cells in adult stomach (Shinohara et al., 2010). In addition, BMP signaling has an inhibitory effect on gastric Lgr5^+^ stem cells as the combination of inflammation leads to enhanced cell proliferation and development of metaplasia and dysplasia in BMP receptor Bmpr1a knockout mice (Ye et al., 2018). Moreover, Hedgehog signaling plays a crucial role in regulation of cell proliferation of the gastric epithelium. Parietal cell-specific knockout of Sonic Hedgehog (Shh) in the neck region led to a high gastrin level, which subsequently increases increased Indian Hedgehog (Ihh) and Wnt signaling and causes hyperproliferation in the pit epithelium (Feng et al., 2014; Xiao et al., 2010). Wnt signaling also plays an important role in the gastric epithelial homeostasis (Leushacke et al., 2017; McCracken et al., 2017; Sigal et al., 2017).

### Intestinal epithelium

The intestine, composed of the small and large intestine, is the place for food digestion, nutrient absorption and hormone secretion. The small intestine is divided into the duodenum that links to stomach, jejunum and ileum that is connected to the large intestine via cecum. The small intestinal epithelium contains numerous crypt-villus structures (Fig. 1b). In the protruding villus, the epithelium contains the mature functional cells of enterocytes, goblet cells, enteroendocrine cells and tuft cells, while the invaginated cryptic epithelium is mainly consisted of immature progenitors, transient amplifying (TA) cells and intestinal stem cells (ISCs) (Barker, 2014; Clevers, 2013; Qi and Chen, 2015; Scoville et al., 2008). At the base of the crypt, the Lgr5^+^ stem cells are intermingled with Paneth cells. Whether there are reserve stem cells at the position 4 is still a matter of dispute (Munoz et al., 2012; Scoville et al., 2008). The large intestine is comprised of the colon and rectum. The single cell-layered intestinal epithelium is a rapid self-renewing tissue, which is driven by the Lgr5^+^ stem cells (Beumer and Clevers, 2016; Wang and Chen, 2018). Lgr5^+^ stem cells possess the ability of self-renewing while generating TA cells (Barker et al., 2007). TA cells migrate upwards to the protruding villus and gradually differentiate into the absorptive enterocytes or secretory cell lineages including enteroendocrine cells, goblet cells and tuft cells. The large intestinal epithelium has only the crypt structure, but no villus. But it has similar cell types as the small intestine except that Paneth cells are missing and their functions could be substituted by Paneth-like cells (Wang et al., 2020).

Like in the stomach, the knowledge of the homeostatic regulation of the intestinal epithelium came from the model organisms such as mouse and fly. Several signaling pathways such as Wnt, Notch, epidermal growth factor (EGF), Hippo and BMP have been shown to control the homeostasis of the intestinal epithelium. Wnt signaling has been demonstrated to be essential in maintaining stem cells and driving proliferation of stem and progenitor cells (Clevers et al., 2014; Van Camp et al., 2014). Inactivation of the downstream Wnt signaling mediator TCF4 causes ISC loss (Korinek et al., 1998), whereas overexpression of Wnt antagonist Dkk1 leads to crypt loss (Kuhnert et al., 2004; Pinto et al., 2003). Notch signaling is also important for the intestinal epithelium homeostasis as disruption of Notch signaling causes loss of proliferating progenitors and accumulation of secretory lineages (Jensen et al., 2000; van Es et al., 2005). EGF ligands execute mitogenic effects on ISCs (Gregorieff et al., 2015), and loss of the EGF receptor negative regulator Lrig1 results in the expansion of the intestinal crypts and carcinogenesis (Powell et al., 2012; Wong et al., 2012). BMP signaling suppresses stem cell signature gene expression, and deletion of the BMP type I receptor BMPR1A in the intestinal epithelium can lead to intraepithelial neoplasia (Qi et al., 2017). In accordance, the overexpression of the BMP inhibitors Noggin or Gremlin causes formation of ectopic crypts and epithelial hyperplasia (Davis et al., 2015; Haramis et al., 2004). Deletion of intestinal epithelial Ihh results in the disappearance of the muscularis mucosae, high Wnt signaling activity and ISC expansion in both the small intestine and colon (Kosinski et al., 2010). In addition, recent studies show that Hippo pathway also plays a role in ISC proliferation as the Hippo signaling mediators YAP/TAZ promote ISC proliferation, epithelial regeneration and suppress goblet and Paneth cell differentiation (Gregorieff et al., 2015; Imajo et al., 2015).

## Generation of 3D human GI organoids

**Fig. 2:**
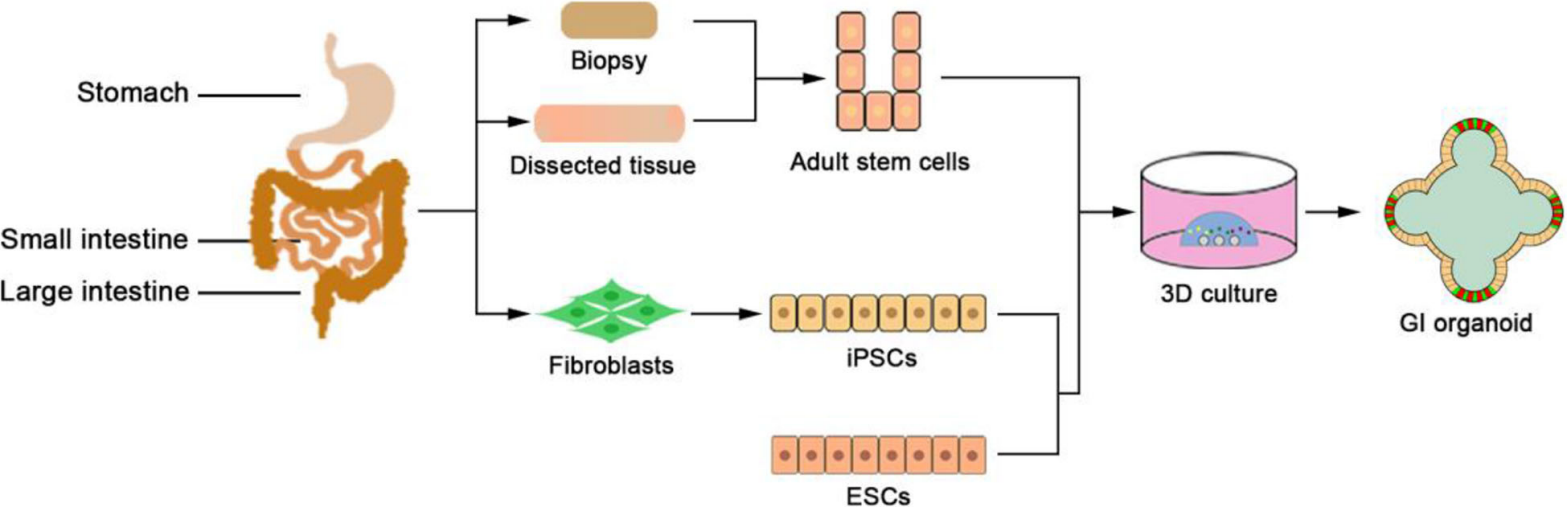
Schematic presentation of the generation of 3D human GI organoids. Adult stem cells are isolated from endoscopic biopsies and surgically dissected tissues. Adult stem cells or pluripotent stem cells are embedded in 3D matrices to generate organoids

Increasing understanding of epithelial homeostasis in the stomach and intestine greatly paves the way for 3D culture of the GI epithelium. Human gastric organoids and intestinal organoids can be successfully generated from the tissue containing adult stem cells and from pluripotent stem cells (Clevers, 2016) (Fig. 2). The first successfully established organoids from the human GI tract is colonic organoids (Jung et al., 2011; Sato et al., 2011). EPHB2 was shown to label colonic stem cells, which could be expanded in vitro as an undifferentiated and multipotent population. By far, organoids have been successfully generated from human gastric epithelium and intestinal epithelium, or organoids with the GI structures and properties can be derived from embryonic stem cells and induced pluripotent stem cells (Barker et al., 2010; Jung et al., 2011; McCracken et al., 2017; McCracken et al., 2014; Sato et al., 2011; Spence et al., 2011).

### Generation of human GI organoids from tissues

#### General procedure

Various protocols have been reported for human GI organoid derivation and culture (Bertaux-Skeirik et al., 2019; Fujii et al., 2015; Hahn and Yoo, 2019; Sato et al., 2011). Here, a general procedure is described. After samples are collected from patients and washed with HBSS, the upper muscular layer and connective tissues are removed by scissors and tweezers. Primary digestive tissues are cut into small pieces and treated with 2 mM EDTA for 30 min on ice, and epithelial cells or cell aggregates are isolated by vigorous continuous suspension. Single stem cells can be obtained by FACS sorting. If the mechanical method is not sufficient for stem cell isolation, mild proteases such as dispase or collagenase can be used for tissue digestion. Next, epithelial cell mixtures or single stem cells are embedded in Matrigel. After Matrigel solidification, organoid culture medium is added. The medium is changed every three to 5 days, and 3D organoid structures can be observed within 1 week. GI organoids can undergo sequential passaging and cryopreservation. They are able to maintain their microscopic appearance and genome stability during long-term culture.

#### Culture matrices

In vitro expansion of organoid requires suitable matrices creating a 3D condition as well as the appropriate medium providing necessary niche factors. Currently, culture of most organoids depends exclusively on the extracellular matrix-based hydrogels, such as Matrigel, basement membrane extracts and different types of collagens (Feng et al., 2009; Sato et al., 2009). Matrigel is the most widely used matrix to simulate the extracellular matrix in organoid culture. Extracted from mouse sarcoma, Matrigel is enriched in extracellular matrix proteins such as collagens, laminin, heparan sulfate proteoglycans and others (Hughes et al., 2010). With the supplement of proper culture medium and growth factors, stem cells embedded in Matrigel can undergo continuous self-renewal and differentiation.

#### Culture medium

In addition to suitable 3D matrices, suitable culture media are critical for successful expansion of GI organoid. Based on what we have learnt from the regulation of human GI epithelium homeostasis and successful culture of mouse GI organoids, culture media for human GI organoids have been formulated (Table 1). These recipes have subtle difference. All culture media contain EGF, BMP antagonist Noggin and Wnt agonist R-spondin1. While the medium supplemented with these factors is sufficient for the culture of murine small intestinal organoids (Sato et al., 2009), more factors are needed to culture human GI organoids.

**Table 1:** Culture media for human GI organoids

Organ	Source of organoids	Basal medium	EGF	Noggin	R-spondin1	Wnt3a	Gastrin	FGF10	Nicotinamide	A-83-01	SB202190	PGE2	Reference
Stomach	Corpus	+^**a**^	50 ng/ml	10% CM	10% CM	50% CM	1 nM	200 ng/ml	Facultative	2 μM	–	–	(Bartfeld et al., 2015)
	Corpus and antrum	+	20 ng/ml	150 ng/ml	25% CM	50% CM	10 nM	150 ng/ml	10 mM	1 μM	2 μM	–	(Schlaermann et al., 2016)
	Fundus	+	50 ng/ml	100 ng/ml	20% CM	50% CM	1 nM	200 ng/ml	10 mM	–	–	–	(Bertaux-Skeirik et al., 2015)
Intestine	Small intestine and colon	+	50 ng/ml	100 ng/ml	1 μg/ml	100 ng/ml	10 nM	–	10 mM	500 nM	10 μM	–	(Sato et al., 2011)
	Small intestine and colon	+	-^**b**^	100 ng/ml	1 μg/ml	50% CM	10 nM	–	-^**b**^	500 nM	-^**b**^	–	(Fujii et al., 2018)
	Colon	+	20 ng/ml	100 ng/ml	1 μg/ml	50% CM	1 μg/ml	–	10 mM	500 nM^**c**^	10 μM	10 nM	(Jung et al., 2011)
	Colon	+^**a**^	50 ng/ml	100 ng/ml	10% CM	50% CM	10 nM	–	–	500 nM	10 μM	–	(Fujii et al., 2019)
Basal medium: Advanced DMEM/F12 + Penicinin/Streptomycin (1X) + Glutamine (2 mM) + N2 (1X) + B27 (1X) + N-acetylcysteine (1 mM) + HEPES (10 mM)													

Typically normal organoid and cancer organoid share the same culture medium. ^**a**^N2 is absent in basal medium. ^**b**^EGF, Nicotinamide and SB20190 is replaced by 50 ng/ml FGF-2 and 100 ng/ml IGF-1. ^**c**^Another TGF-β inhibitor LY2157299 is used instead of A83–01. *CM* Conditional medium

As mentioned above, Wnt signaling is crucial for epithelia homeostasis and tissue regeneration in the GI tract (Clevers et al., 2014; Van Camp et al., 2014). R-spondin1 and Wnt3a are included in all the expansion media for human GI organoids, indicating a greater demand of Wnt signaling activity to promote human organoid growth. In addition, other growth factors and small molecules have been demonstrated to be important for maintenance of human GI organoids. FGF10 is essential for the generation of gastric organoids, consistent with its function in promoting cell proliferation and inhibiting differentiation (Speer et al., 2012). Gastrin, which acts through the cholecystokinin G-protein-coupled receptor-phospholipase C-calcium pathway, has a mitogenic effect on gastric cells (Yassin, 1999). Nicotinamide, which is also known as vitamin B3, suppresses sirtuins activity and promotes human colonic organoid forming efficiency (Jung et al., 2011). The TGF-β/Activin receptors ALK4/5/7 inhibitor A83–01 and the p38 inhibitor SB202190 significantly improve the plating efficiency and synergistically increase the number of passages of the human colonic organoids, in accordance with the inhibitory effect of TGF-β signaling on intestinal epithelial cells (Jung et al., 2017). Prostaglandin E2 (PGE2) is found necessary for human GI propagation (Jung et al., 2011), which probably functions via cAMP-mediated blockage of anoikis and stimulation of MAP kinase signaling (Goessling et al., 2009; Jiang et al., 2017). It is worth mentioning that ROCK1 inhibitor Y-27632 is essential to avoid anoikis in the early culture time (Bertaux-Skeirik et al., 2015; Sato et al., 2011). We have also reported that the non-muscle-myosin-II inhibitor blebbistatin can enhance the survival and expansion of the organoids derived from single murine Lgr5^+^ intestinal stem cells through activation of the Akt signaling (Zhao et al., 2015).

The beauty of organoids is the mimic of tissue structures and functions by sustaining the self-renewal of tissue stem cells while retaining the diversity of tissue cell types. To sustain the continuous self-renewal of stem cells in vitro and long-term culture of organoids, strong mitogenic signals such as Wnt and EGF are needed. However, excessive activation of Wnt and EGF signaling could keep GI organoids in an undifferentiated state, and certain kinds of differentiated cells are missing. For example, parietal cells are missing in the gastric organoids cultured with the medium containing 20 ng/ml EGF and 50% Wnt3a conditional medium (Schlaermann et al., 2016). Furthermore, for mechanistic studies and possible medical applications, it is also important to obtain homogeneous population of certain cell types. To achieve this, various conditions have been applied to induce cell differentiation. Table 2 lists the conditions to induce cell differentiation and major differentiated cell types in the GI organoids. The trade-off, however, is that in the differentiation medium organoids can only be maintained for a short time. Recently Sato and colleagues developed a culture medium that can improve the culture efficiency and maintains long-term multi-differentiation capacity of human small intestinal organoids (Fujii et al., 2018). EGF, nicotinamide and the p38 inhibitor SB202190 were replaced with IGF-1 (insulin-like growth factor-1) and FGF-2. This study may bring inspiration for the optimization of the human GI organoid culture in the future.

**Table 2:** Differentiation media of human GI organoids

Organ	Source of organoid	Difference from expansion medium	Differentiated cells	References
Stomach	Corpus	+Nicotinamide/−Wnt3a	Gland lineage cells/pit lineage cells	(Bartfeld et al., 2015)
	Corpus and antrum	-Wnt3a, −R-spondin1	MUC5AC-producing pit mucous cells	(Schlaermann et al., 2016)
Intestine	Small intestine and colon	-Wnt3a, −Nicotinamide, −SB202190	Goblet cells and enteroendocrine cells	(Sato et al., 2011)
	Colon	-Wnt3a, −Nicotinamide, −SB202190, −PGE2, +DAPT^**a**^	Enterocytes, goblet cells and enteroendocrine cells	(Jung et al., 2011)

^a^10 μM gamma-secretase inhibitor DAPT is added to inhibit Notch signaling

### Generation of human GI organoids from pluripotent stem cells

As pluripotent stem cells (PSCs), including embryonic stem (ES) cells and induced pluripotent stem (iPS) cells, have the capacity of giving rise to all cell types, and are easily manipulated by gene-editing, they have been widely used for investigation of human development and pathogenesis. However, their cell type homogeneity limits their applications. Since organoids can more accurately reflect the multicellular interactions in physiological and pathological conditions, PSC-derived organoids then become great models for understanding of human organogenesis and pathogenesis (Clevers, 2016; Eiraku et al., 2011; Lancaster and Knoblich, 2014; Li et al., 2016; Rowe and Daley, 2019; Spence et al., 2011).

**Fig. 3:**
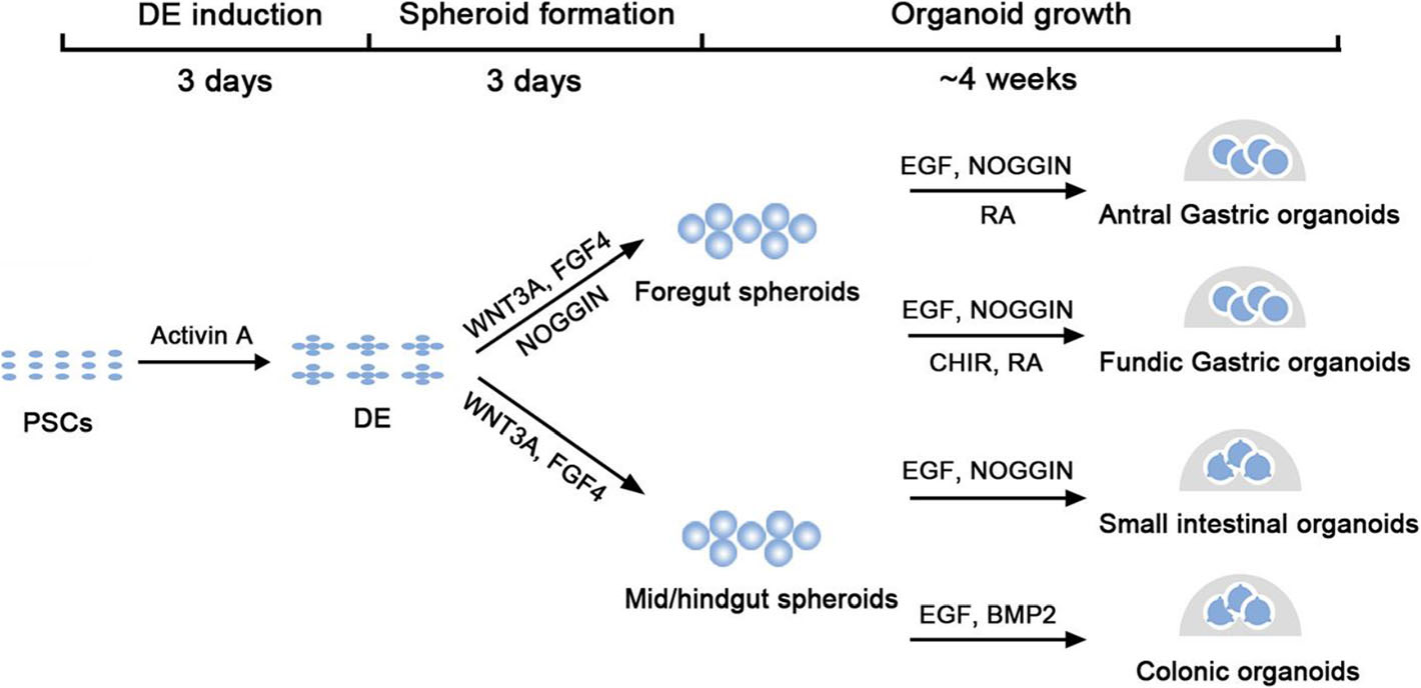
Schematic representation of the in vitro culture system to direct the differentiation of pluripotent stem cells into 3D human GI organoids. Retinoic acid (RA) is used to induce to posterior foregut fate. DE: definitive endoderm

Studies of embryonic development in model organisms have made successful efforts to direct the differentiation of PSCs into specific tissue cell types in vitro. By mimicking development processes, tissue-specific organoids can be induced (Fig. 3). The major process usually includes germ layer induction, tissue-specific spheroid formation and organoid specification. As the digestive system develops from endoderm, endoderm patterning is proved to be essential for the GI organoids induction (Broda et al., 2019; Hannan et al., 2013; McCracken et al., 2017; McCracken et al., 2014; Spence et al., 2011; Uchida et al., 2017). Differentiation into the definitive endoderm from human ES cells and iPSCs is achieved by activin A treatment for three consecutive days with increasing concentrations of defined fetal bovine serum (D'Amour et al., 2005). After definitive endoderm induction, cells are cultured in the medium containing 2% defined fetal bovine serum with a combination of growth factors to trigger foregut (Wnt3a, FGF4, Noggin) or mid/hindgut (Wnt3a, FGF4) differentiation. To induce a posterior fate in foregut endoderm, retinoic acid is needed in the last day. After 2–4 days treatment with these factors, floating 3D spheroids are formed. The spheroids are resuspended in Matrigel and overlaid with corresponding organoid culture medium for the culture of adult stem cells-derived GI organoids. Then, spheroids develop into organoids in a staged manner that is notably similar to fetal gut development. The resulting organoids have notable cell type complexity, structure and function similar to their in vivo counterparts. For instance, PSC-derived human gastric organoids contain a complex epithelium with glandular architecture and surrounding mesenchymal cells (McCracken et al., 2014). PSC-derived human intestinal organoids exhibit a columnar epithelium with villus-like structures protruding into the lumen (Spence et al., 2011).

Generation of GI organoids from PSCs provides a way to generate multicellular 3D structures mimicking native human tissues. In particular, using various established methods, iPS cells can be handily obtained from different types of cells from normal or pathological tissues (Karagiannis et al., 2019). In addition, PSCs can be easily manipulated with gene editing and other genetic modifications. Therefore, PSC-derived organoids serve as great models for the study on the mechanisms underlying human digestive diseases and facilitate drug discovery. Moreover, the ability to generate autogenous tissues should greatly benefit regenerative medicine. However, it is worth noting that PSC-derived GI organoids show limited cell maturation with characteristics resembling fetal tissues rather than adult ones (McCracken et al., 2014; Spence et al., 2011).

### Air-liquid method to generate GI organoids

In addition to the classical 3D culture method in supportive matrices, air-liquid interface culture is also used to generate GI organoids via improved oxygenation in vitro (Ootani et al., 2009; Wang et al., 2015). Schmidt et al. reported the first air-liquid culture in which human nasal cells could be maintained in an air-liquid interface culture with the production of ciliated cells (Schmidt et al., 1996). Then, Kuo and colleagues improved this system for long-term organotypic intestinal culture (Ootani et al., 2009). In their system, an inner dish with a permeable membrane bottom is coated with collagen, overlaid with small intestinal minces embedded in collagen, and the inner dish is placed to an outer dish supplemented with the culture medium. The level of the medium in the outer dish is below the cellular gel layer to allow air exposure of the cellular layer. In a few days 3D intestinal spheres are produced with a polarized epithelial monolayer containing various intestinal cell types. In this system, growth of the epithelial spheres requires myofibroblasts, thus allowing the study on the interaction between epithelial cells and niche cells. In 2015, using the air-liquid interface culture system, Wang et al. found that the “ground state” human intestinal stem cells that possess higher clonogenicity could differentiate and gave rise to columnar intestinal epithelium with villus-like structures (Wang et al., 2015). The advantage of the air-liquid approach is the ability to perform physiologically relevant studies under controlled conditions.

## Application of human GI organoids

**Fig. 4:**
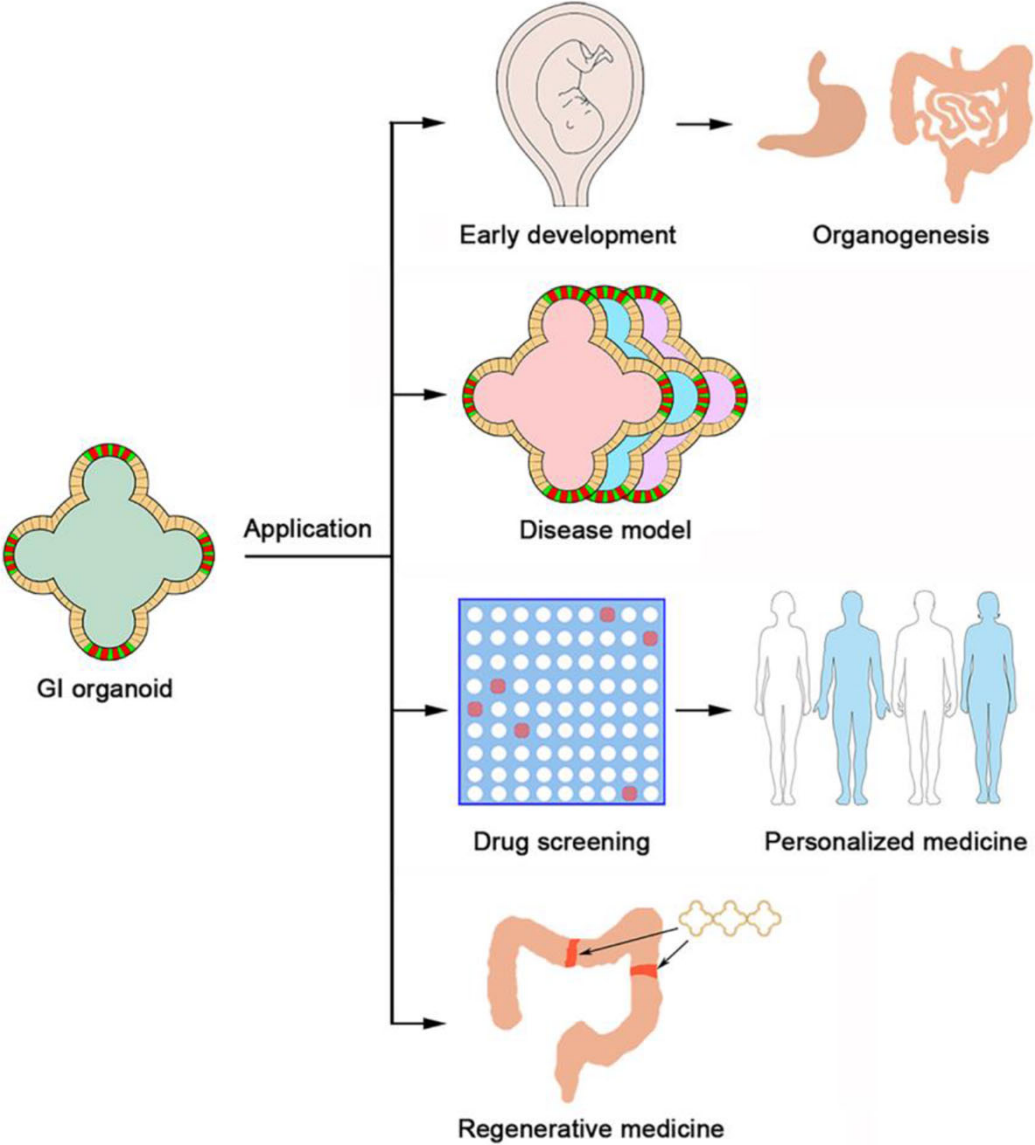
Application of human GI organoids. GI organoids can be used for investigating early development, modeling different digestive diseases, screening drugs and regenerative medicine

One important feature of organoids is their great experimental operability. Many standard experimental methods conducted on cell lines can be applied to organoids, such as DNA transfection, virus infection, gene knockout, mRNA and protein isolation as well as immunohistochemistry and immunofluorescence analysis (Drost et al., 2015; Lancaster and Knoblich, 2014). This makes human GI organoids to be excellent experimental tools for biomedical research and clinical applications (Fig. 4).

### Elucidation of the mechanisms underlying human GI tract development

In the past, due to the difficulty in accessing human embryonic material, study on early morphogenesis is severely limited, and as a result, there are many gaps in our understanding of the molecular mechanisms underlying human embryonic development and organogenesis. Organoids provide a solution. As a simple and handy system, GI organoids share similar features with in vivo digestive tissue. For instance, during the maturation process of the human small intestinal organoids, primary intestinal stem cells existing in crypts can generate de novo lumen-containing spheroids with crypt-like buds (Date and Sato, 2015; Sato et al., 2009). As GI organoids can be generated from PSCs by mimicking embryonic development, in turn they allow the visualization and analysis of human stomach and intestinal development in vitro (Cruz-Acuna et al., 2017; Hannan et al., 2013; McCracken et al., 2014; Spence et al., 2011).

Using PSC-derived gastric organoids, McCracken et al. found that while Wnt and FGF induce the morphogenesis of gut tube-like structures, inhibition of BMP signaling induces expression of the foregut genes (McCracken et al., 2014). In a follow-up study, the same group successfully generated gastric organoid containing fundic epithelium from PSCs and identified key events in embryonic fundus development (McCracken et al., 2017). Upon activation of Wnt signaling at the posterior foregut stage, the gene expression profile of gastric organoid progenitors shifts from antral to fundic identity, indicating the essential role of Wnt/β-catenin signaling in fundus specification. They further reported that MEK/ERK signaling inhibits parietal cell specification.

Moreover, Munera et al. reported that a conserved BMP-HOX axis is required to establish posterior identity and activating BMP signaling in PSC-derived gut spheroids generates human colonic organoids that retain colonic identity after transplantation (Munera et al., 2017). These results revealed the importance of BMP signaling in endoderm patterning during intestinal development. Using PSC-derived gastric organoids, retinoic acid was also shown to be important for posterior foregut specification (McCracken et al., 2014), which was consistent with the role of retinoic acid signaling in development of posterior-foregut-derived organs (Cunningham and Duester, 2015).

### Disease modeling

Another major application of GI organoids is as a model to elucidate human-specific digestive disease mechanisms. Due to the unique identity of organoids reflecting key structural and functional properties of organs, there is an unprecedented opportunity to take GI organoids as digestive disease models (Clevers, 2016; Dutta et al., 2017).

Cancer is the second leading cause of death worldwide, and gastric and colorectal cancers are extremely common (Sengupta and Honey, 2018). In the past, most studies on carcinogenesis were based on cancer cell lines and animal models. As cancer formation occurs in a multi-cellular environment, organoids can nicely fill the gap. As soon as the culture method for GI organoids from adult stem cells was developed, culture of GI cancer organoids from primary colorectal, gastric cancers as well as liver and pancreatic cancers became reality (Bartfeld et al., 2015; Boj et al., 2015; Broutier et al., 2017; Fujii et al., 2019; Jung et al., 2011; Prior et al., 2019; Sato and Clevers, 2013; Sato et al., 2011). Interestingly, culture of GI cancer organoids has a remarkably high success rate compared with establishment of primary cancer cell lines and patient-derived xenograft (PDX) models (Aboulkheyr Es et al., 2018; Weeber et al., 2017). The organoids derived from human colorectal cancer metastases also preserve genetic diversity as the original metastases (Weeber et al., 2015). Using CRISPR/Cas9 technology to target the most commonly mutated colorectal cancer genes in normal human intestinal organoids, GI organoids can be used to mimic the early stages of cancer formation step by step (Drost et al., 2015; Fujii et al., 2019; Matano et al., 2015).

In addition to being used in cancer studies, GI organoids can also be used to model other diseases. Cystic fibrosis is a common genetic malady caused by defects in the cystic fibrosis transmembrane conductance regulator (CFTR) gene and causes malfunctions in multiple organs including the GI tract (Ooi and Durie, 2016). A phenotype of cystic fibrosis patients is loss of swelling response of GI epithelial cells to increased cAMP resulted from CFTR mutations. By correcting the CFTR gene mutations with CRISPR/Cas9 editing in patient-derived intestinal organoids, Schwank et al. was able to restore the cAMP response and cell functions (Schwank et al., 2013). Using PSC-derived human intestinal organoids and neural crest cells, Workman et al. were able to recapitulate the development of the enteric nervous system and model Hirschsprung’s disease caused by PHOX2B mutation (Workman et al., 2017). The mucous layer of human digestive system constructs the first line of defense against pathogen infection. Owing to the early manipulation of organoid-pathogen coculture, the GI organoids culture system provides a splendid platform to study the host–microbe interactions. Till now, a wide variety of infection models have been established for pathogens, such as *Helicobacter pylori*, *Cryptosporidium parvum*, *Salmonella enterica* and *Clostridium difficile* (Bartfeld, 2016; Hill and Spence, 2017). Ulcerative colitis (UC) is an idiopathic chronic inflammatory bowel disease characterized by persistent inflammation that begins in the rectum. Recently, Sato and his colleagues reported a specific somatic mutation pattern in the organoids derived from UC patients that is associated with affected IL-17 signaling (Nanki et al., 2020). This work highlights a power of organoids in identifying genetic changes in adaptation to adverse microenvironments and demonstrates their potential impact to understand UC pathogenesis.

### Drug screening and personalized medicine

Organoids can faithfully reflect the properties of the original tissues, similarly organoids derived from cancers also possess the complexity and heterogeneity of the cancers of origin. Therefore, cancer organoids (tumoroids) are an ideal system for anti-cancer drug screening with two apparent advantages: 1) preservation of the most characteristics of the cancer of origin; 2) the feasibility and economy to maintain and amplify them in a large scale (Weeber et al., 2017). Cancer organoid biobanks that collect organoids from individual patients provide a great platform for cancer research and drug screening. Various GI cancer organoid biobanks have been established (Fujii et al., 2016; Saito, 2019; van de Wetering et al., 2015; Vlachogiannis et al., 2018; Yan et al., 2018; Yao et al., 2020). Drug sensitivity of tumor organoids is proven to be consistent with the cancer molecular subtypes. In 2015, van de Wetering et al. generated a colorectal cancer organoid biobank from 20 patients (van de Wetering et al., 2015). They tested 83 specific compounds and found that the organoids carrying TP53 mutants were insensitive to MDM2 inhibition and the ones with RAS mutants resistant to EGF receptor inhibition. It is noteworthy that RNF43 mutants were found to be extremely sensitive to Wnt inhibitors. Analysis of another colorectal cancer organoid biobank containing 55 different organoids that represent different histological subtypes and clinical stages revealed that niche factors-dependent growth of cancers is mainly related to cancer progress from adenoma to carcinoma (Fujii et al., 2016). Recently, using a rectal cancer biobank with 80 tumor organoids, Yao et al. demonstrated that chemoradiation responses in organoids were correlated with the clinical outcome (Yao et al., 2020), further demonstrating the important application of patient-derived organoids in the clinic. Moreover, assessment of gastric cancer organoid biobanks that was consisted of known molecular subtypes of gastric cancers uncovered that these organoids exhibited the similar characteristics to the corresponding primary tissues and also demonstrated that these organoid biobanks can be used for high-throughput drug screening (Seidlitz et al., 2019; Yan et al., 2018).

Another important application of patient-derived organoids is a rapid screen and test of drugs for personalized medicine. As organoids derived from resection or biopsy can be fast expanded, the sensitivity to therapeutic drugs can be tested in a short period of time to guide disease treatment in a personalized manner. A proof-of-concept clinical example is the identification of a drug with the intestinal organoids derived from a cystic fibrosis patient carrying a rare CFTR mutation, and the drug named Kalydeco was shown to be effective (Saini, 2016).

In addition, GI organoids have a potential in regenerative medicine (Aboulkheyr Es et al., 2018; Rossi et al., 2018). At present the shortage of donor organs remains a serious problem. The organoids derived from patient iPSCs can supply autologous cells or tissues for transplantation without immunological rejection. Furthermore, organoids derived from gene-edited iPSCs could be used to replace the dysfunctional tissues with genetic mutations. Although no clinical applications have been reported yet, relevant studies have been conducted in animal models. Mouse colonic organoids or fetal enterospheres were able to regenerate injured colonic mucosa (Fordham et al., 2013; Yui et al., 2012).

## Limitations and perspectives

While human GI organoids have been shown to be a superb platform for in vitro biomedical research and drug testing, some nonnegligible limitations still exist. Matrigel is not a well-defined matrix, which greatly hampers the application of organoid techniques to regenerative and translational medicine (Hughes et al., 2010). For this reason, well-defined synthetic matrices should be alternatives. Polyethylene glycol (PEG)-based hydrogels together with other defined factors have been reported to successful culture of murine and human intestinal organoids (Gjorevski et al., 2016). Subsequently, a synthetic PEG-4MAL hydrogel was used to support in vitro generation of intestinal organoids from human ESC- and iPSC-derived 3D spheroids without Matrigel (Cruz-Acuna et al., 2017).

GI organoids can’t completely reflect pathophysiological conditions as some rare epithelial cell types may be missing. For instance, secretin^+^ enteroendocrine cells were lost in small intestinal organoids (Basak et al., 2017; Beumer et al., 2018). Moreover, current organoid culture systems are developed mainly for expansion of epithelial cells, and hence niche cells such as stromal cells, immune cells, and vasculature endothelial cells are missing. As niche cells play an important role in controlling tissue homeostasis, much effort is needed to include niche cells for organoid culture to better mimic the pathophysiological conditions. This is extremely important for us to have a better understanding of gastric ulcer and inflammatory bowel disease.

Current organoid culture systems yield a spherical structure with an enclosed lumen whose apical domain face toward the organoid lumen. Unlike in the normal GI architecture having the apical domain facing to the GI tract lumen, the apical domain of organoids is not easily accessed by drugs, toxins, microorganisms and other materials, which seriously impede the pathogen-host cell interaction studies and relevant research. 3D spatial structure brings much difficulties to high-throughput imaging. To overcome these limitations, other alternative systems have been developed. For instance, Wang et al. designed a collagen-coated polydimethylsiloxane (PDMS) scaffold similar to the crypt or crypt-villus structure (Wang et al., 2017; Wang et al., 2018). After loading cells on the scaffold, they obtained self-renewing 3D intestinal epithelium with open luminal surface and cells of different lineages.

Furthermore, 2D monolayer culture may also serve as a substitution for 3D GI organoids. Due to rapid cell death and stem cell loss, monolayer culture of GI epithelial cells was not successful until recently. With a thin extracellular matrix-based coating, several simple and economical 2D monolayer culture systems have been established (reviewed in (Liu and Chen, 2018)). The culture derivatives can recapitulate most of the features of 3D-cultured organoids and in vivo tissue. Exposure of the surface in 2D culture provides a suitable system to investigate the interactions between different cell types, the dynamics of resident stem cells and the pathogen-host cell interaction. It is worth mentioning that the present epithelial monolayer cultures have not achieved long-term propagation.

Moreover, various growth factors are supplemented in the culture medium to maintain organoid growth. The complex nature and high cost limit their application on a large scale. Recently, we have established a growth factor-free culture system of murine intestinal organoids with two small molecules (Li et al., 2018), lighting a hope in this direction. Similarly, small molecules should be very useful to achieve a better differentiation to specific cell types as shown in murine cell differentiation (Yin et al., 2014).

As current culture medium is mainly designed for the expansion of the epithelial cells, a considerable challenge for cancer organoid culture is the overgrowth of the normal cells (Weeber et al., 2017). Long-term culture of cancer organoids may lose heterogeneity, which may greatly hampers application of cancer organoid. Therefore, different culture recipes are needed for different subtypes of cancer organoids.

Despite existing challenges, the research of human GI organoids still holds a promising future. GI organoids and organoids resembling other tissues can be assembled into assembloids with complex structures, which can shed new light on tissue or organ interactions (Marton and Pasca, 2019). With the help of organoids, understanding of pathogenesis of complicated diseases and drug development will be accelerated.
